# Dab1 Contributes to Angiotensin II-Induced Apoptosis via p38 Signaling Pathway in Podocytes

**DOI:** 10.1155/2017/2484303

**Published:** 2017-06-05

**Authors:** Zhao Gao, Xinghua Chen, Kai Zhu, Ping Zeng, Guohua Ding

**Affiliations:** Division of Nephrology, Renmin Hospital of Wuhan University, 238 Jiefang Rd., Wuhan, Hubei 430060, China

## Abstract

Numerous studies have found that angiotensin II (Ang II) participates in podocyte apoptosis and exacerbates progression of end-stage kidney disease (ESKD). However, its underlying mechanism remains largely unexplored. As a homolog of Drosophila disabled (Dab) protein, Dab1 plays a vital role in cytoskeleton, neuronal migration, and proliferation. In the present study, our data revealed that Ang II-infused rats developed hypertension, proteinuria, and podocyte injury accompanied by Dab1 phosphorylation and increased reelin expression in kidney. Moreover, Ang II induced podocyte apoptosis in vitro. Dab1 phosphorylation and reelin expression in podocytes were increased after exposure to Ang II. Conversely, Dab1 small interfering RNA (siRNA) exerted protective effects on Ang II-induced podocyte apoptosis, resulting in decreased p38 phosphorylation and reelin expression. These results indicated that Dab1 mediated Ang II-induced podocyte apoptosis via p38 signaling pathway.

## 1. Introduction

As terminally differentiated cells, podocytes play a crucial role in establishing the integrity and selective permeability of the glomerular filtration barrier [1–3]. Accumulating evidence has shown that podocyte injury is associated with proteinuria and several glomerular diseases [4–7]. As an important risk factor for the initiation and progression of chronic kidney diseases [8, 9], angiotensin II (Ang II) exerts hemodynamic effects on renal tissue and a direct effect on podocyte injury. Recent studies have shown that Ang II can induce podocyte apoptosis both in vivo and in vitro [10–12]. However, the exact molecular mechanism of Ang II-induced podocyte apoptosis remains largely unexplored.

Disabled-1 (Dab1), a homolog of the Drosophila disabled protein, consists of three main domains as follows: C-terminal serine/threonine-rich region, N-terminal protein interaction/phosphotyrosine binding domain, and a tyrosine-rich region. Dab1 located in cytoplasm is an adaptor protein associated with neuronal migration and polarization [13]. When reelin, a secreted extracellular matrix glycoprotein, or apoE binds to very low-density lipoprotein receptor (VLDLR) and/or apolipoprotein receptor 2 (ApoER2), Dab1 interacts with the SH2 domains of Src, Fyn, and Abl, leading to Dab1 tyrosine phosphorylation and activation of downstream signaling pathways, such as Crk-C3G-Rap1-cadherin pathway and PI3K-Akt pathway [14].

Our previous study has indicated that c-Abl upregulation promotes podocyte apoptosis upon exposure to Ang II via Akt signaling [12]. In the preexperiment, we found that Dab1 and reelin protein were expressed in rat and podocytes by western blotting and immunofluorescence assay. We hypothesized that Dab1 participated in podocyte apoptosis and aimed to examine the role of Dab1 in Ang II-induced podocyte apoptosis and signal transduction.

## 2. Materials and Methods

### 2.1. Animals

A total of 16 male specific-pathogen-free (SPF) Sprague-Dawley rats (weighing 110–140 g) were supplied by the Research Center of Medical Experimental Animals of Wuhan University. Animals were bred in an atmosphere with controlled temperature and humidity under an artificial light cycle. Food pellets and water were available ad libitum. Rats embedded with osmotic minipump (Alzet model 2002 or 2004, CA) were randomly subjected to normal saline infusion or Ang II infusion at a dose of 400 ng/kg/min for 4 weeks. Tail cuff plethysmography was used to measure systolic blood pressure at different time points (weeks 1, 2, 3, and 4). Rats were individually placed into metabolic cages for 24 h to collect urine, and urinary albumin was determined at the above-mentioned time points (weeks 1, 2, 3 and 4). Urinary albumin was determined with a competitive ELISA kit (Shibayagi, Shibukawa, Japan). Animals were sacrificed after 4 weeks. Part of the kidney was fixed in 4% phosphate-buffered paraformaldehyde for pathological analysis of renal damage, and the rest was stored at −80°C prior to further biochemical analysis. Glomeruli were isolated by 177 *μ*m, 125 *μ*m, and 74 *μ*m stainless steel sieves. The experimental procedures and protocols were approved by the Ethical Committee for the Experimental Use of Animals of Renmin Hospital of Wuhan University, Wuhan, Hubei Province, China.

### 2.2. Cell Culture

Conditionally immortalized mouse podocytes were kindly provided by Dr. Peter Mundel (Massachusetts General Hospital, Boston, MA). Podocytes were cultured in RPMI 1640 medium (HyClone, USA) supplemented with 10% heat-inactivated fetal calf serum (Gibco, USA), 100 U/mL penicillin G, 100 mg/mL streptomycin, and 10 U/mL recombinant murine interferon-*γ* (Pepro Tech, Rocky Hill, NJ) at 33°C. Proliferating podocytes were transferred to an incubator at 37°C for 7 days to induce differentiation without interferon. The differentiated cells were stimulated with Ang II (Enzo Life Sciences, Farmingdale, NY) for various durations.

### 2.3. Dab1 siRNA Transfection

Dab1 siRNA transfection was carried out according to the HiPerFect Transfection Reagent Handbook (QIAGEN, Germany). Four Dab1 siRNAs (siRNA sequences: si1 AAGGGAGAACACAAACAGAAA, si3 CAGCGAAGCCACTTTGATAAA, si4 CACTTTGATAAAGAGGTTTAA) were designed and synthesized by QIAGEN (Germany). Briefly, 2 × 10^5^ cells were seeded in a 6-well plate and then transfected with a 100 *μ*L mixture containing 150 ng Dab1 siRNA (or a negative control with scrambled siRNA) and 12 *μ*L of HiPerFect transfection reagent for 48 h.

### 2.4. Double Immunofluorescence Assay

The cell climbing film was fixed in 4% paraformaldehyde at 4°C for 30 min and then blocked in 5% bovine serum albumin at room temperature for 30 min. The films were, respectively, stained with the following antibodies: Dab1 antibody (1 : 50, sc-13981, Santa Cruz Biotechnology), phospho-Dab1 antibody (1 : 25, sc-133293, Santa Cruz Biotechnology), and reelin antibody (1 : 50, sc-25346, Santa Cruz Biotechnology) at 4°C overnight. Subsequently, films were incubated with FITC-conjugated IgG as the secondary antibody at 37°C for 45 min in the dark. The sections were observed under fluorescence microscope (Olympus, Japan).

Paraffin-embedded sections were deparaffinized and treated with 3% H_2_O_2_ at room temperature for 30 min. Antigen retrieval for Dab1, phospho-Dab1, and reelin was carried out in high-pressure citrate buffer (0.01 M, pH 6.0) for 10 min. The sections were blocked with 5% bovine serum albumin for 30 min, incubated with nephrin antibody (1 : 100, sc-32529, Santa Cruz Biotechnology), Dab1 antibody (1 : 50, sc-13981, Santa Cruz Biotechnology), phospho-Dab1 antibody (1 : 30, sc-133293, Santa Cruz Biotechnology), and reelin antibody (1 : 50, sc-25346, Santa Cruz Biotechnology) at 4°C overnight, and then stained with FITC-conjugated IgG at 37°C for 60 min in the dark. All microscopic images were recorded using a fluorescence microscope (Olympus, Japan).

### 2.5. Apoptosis Assay

Podocyte apoptosis in kidney tissue was assessed by TUNEL (Roche, Germany) according to the manufacturer's instructions. Briefly, the paraffin-embedded sections were dewaxed and incubated with 3% H_2_O_2_ for 30 min. Antigen retrieval was performed in high-pressure citrate buffer (0.01 M, pH 6.0) for 10 min. Subsequently, the sections were blocked with 10% goat serum at room temperature for 30 min and incubated with terminal deoxynucleotidyl transferase (TdT) and digoxigenin-11-dUTP at room temperature for 1 h. Next, sections were incubated with streptavidin-biotin-peroxidase-conjugated antidigoxigenin-11-dUTP antibody for 30 min. The negative control was omitted TdT. The podocytes were counted in 3 glomerular cross sections using 10 randomly selected fields. Two consecutive sections were stained with PAS and TUNEL, respectively. The sections were scanned by automatic digital slice scanning system (Leica SCN400F, Germany). Podocytes were identified and counted in PAS section [15, 16]. In the same view, apoptotic podocytes were counted in TUNEL section.

### 2.6. Western Blotting

Glomeruli and podocytes were lysed with RIPA buffer (Beyotime, China) containing protease inhibitor cocktail (Sigma-Aldrich) and PMSF (Beyotime, China) on the ice for 30 min, and then the cell lysates were centrifuged at 12,000 rpm for 10 min at 4°C. Subsequently, the supernatants were mixed with 5x loading buffer and boiled at 100°C for 5 min. The proteins were separated with SDS-PAGE and then electrotransferred onto PVDF membranes (Merck Millipore). The membranes were incubated with primary antibodies (rabbit polyclonal Dab1 antibody, 1 : 50, Santa Cruz Biotechnology, Dallas, TX; rabbit polyclonal phospho-Dab1 antibody, 1 : 50, Santa Cruz Biotechnology, Dallas, TX; mouse monoclonal reelin antibody, 1 : 50, Santa Cruz Biotechnology, Dallas, TX; rabbit polyclonal p38 antibody, 1 : 1,000, Cell Signaling Technology; rabbit polyclonal phospho-p38 antibody, 1 : 1,000, Cell Signaling Technology; rabbit polyclonal ERK antibody, 1 : 1,000, Cell Signaling Technolog; rabbit polyclonal phospho-ERK antibody, 1 : 1,000, Cell Signaling Technology; rabbit polyclonal caspase-3 antibody, 1 : 1000, GeneTex Inc.; rabbit polyclonal bcl-2 antibody, 1 : 1000, GeneTex Inc.; rabbit polyclonal *β*-actin antibody, 1 : 1000, Antgene, Hubei, China) at 4°C overnight. An Alexa Fluor 790-labeled goat anti-rabbit/mouse IgG (1 : 30,000; Jackson ImmunoResearch, USA) was used as secondary antibody, and the blots were visualized with a LI-COR Odyssey Infrared Imaging System.

### 2.7. Statistical Analysis

Data were presented as means ± SD and analyzed with SPSS 17.0. Student's* t* test or one-way analysis of variance was used to compare differences between groups. A *P* value < 0.05 was considered statistically significant.

## 3. Results

### 3.1. Effect of Ang II on Kidney Injury

**Figure 1:**
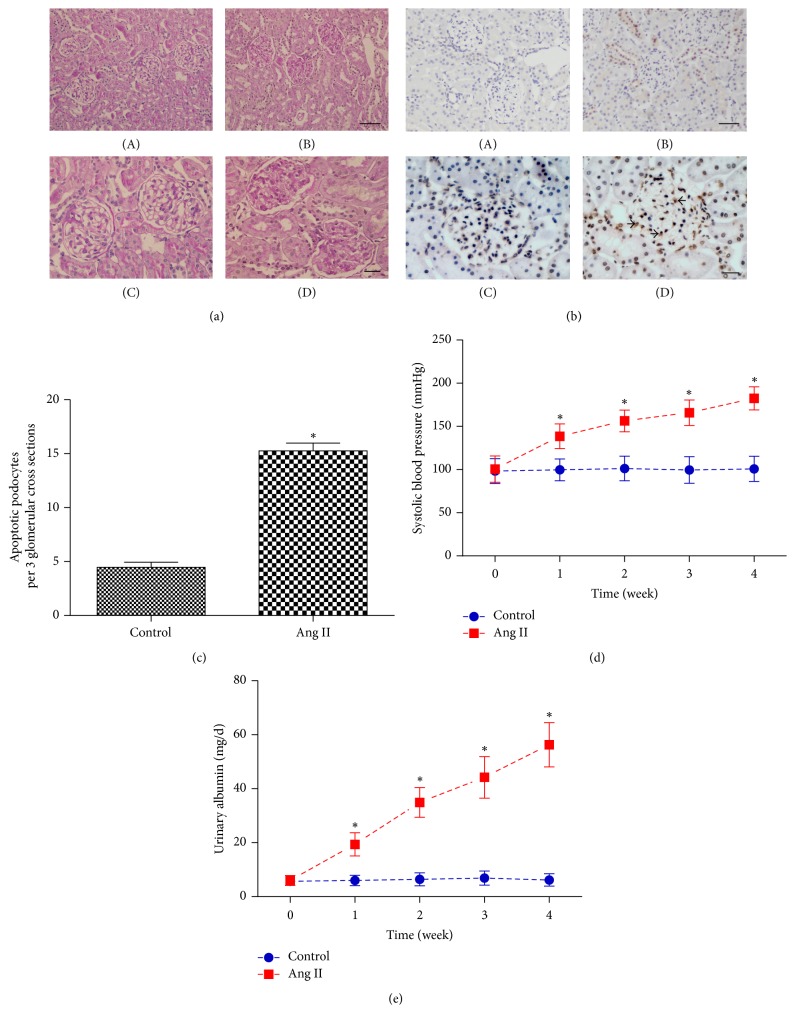
Clinical and pathological changes. (a) Glomerular pathological changes with PAS staining (original magnification ×200, ×400); (A) control group in week 4; (B) Ang II-infused group in week 4. Scale bars, 50 *μ*m. (C) Control group in week 4; (D) Ang II-infused group in week 4. Scale bars, 20 *μ*m (*n* = 6). (b) Apoptotic podocytes of rat kidneys were assessed by TUNEL staining (original magnification ×100, ×400); (A) control group in week 4; (B) Ang II-infused group in week 4; scale bars, 100 *μ*m. (C) Control group in week 4; (D) Ang II-infused group in week 4. The black arrows indicate apoptotic podocytes. Scale bars, 20 *μ*m (*n* = 6). (c) Quantification of apoptotic podocytes. (d) Systolic blood pressure in weeks 1, 2, 3, and 4. (e) 24 h urinary albumin in weeks 1, 2, 3, and 4. ^*∗*^*P* < 0.05 versus control group.

Figure 1(a) exhibits that the manifestation of glomerulus in Ang II infused rats showed cellular proliferation and accumulation of extracellular matrix. The characterization of glomerular damage was consistent with our past observation [10]. Moreover, the percentage of apoptotic podocytes in Ang II-infused rats was significantly increased compared with the control group (Figures 1(b) and 1(c)). Figures 1(d) and 1(e) show that systolic blood pressure and urinary albumin level were increased in Ang II-infused rats compared with controls in weeks 1, 2, 3, and 4.

### 3.2. Effect of Ang II on Reelin/Dab1 Expression in Kidney

**Figure 2:**
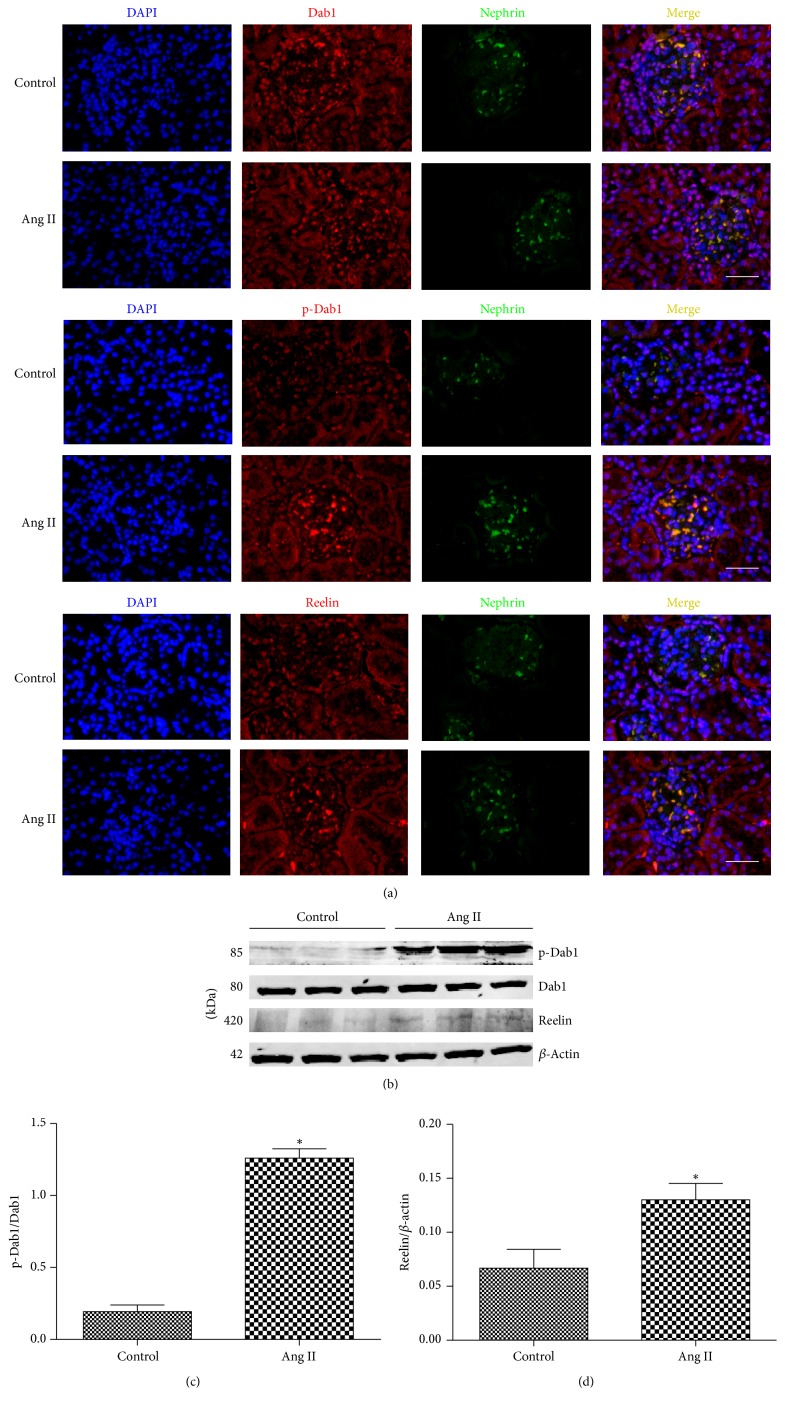
Ang II increases reelin/Dab1 expression in kidney. (a) Double immunofluorescence staining for podocyte marker nephrin and various molecules (Dab1, phospho-Dab1, and reelin) in rat kidney (original magnification ×200); scale bars, 50 *μ*m. (*n* = 6). (b) Western blotting detected the expressions of phospho-Dab1, Dab1, and reelin in kidney of rats infused by Ang II. (c, d) Quantitative analysis of phospho-Dab1 and reelin expression. ^*∗*^*P* < 0.05 versus control group.

Figure 2(a) demonstrates that the reelin expression and phosphorylated Dab1 level in Ang II-infused rats were higher than those in the control group. The results of western blotting were consistent with double immunofluorescence assay (Figures 2(b)–2(d)).

### 3.3. Effect of Ang II on Reelin/Dab1 Expression in Podocytes and Podocyte Apoptosis

**Figure 3:**
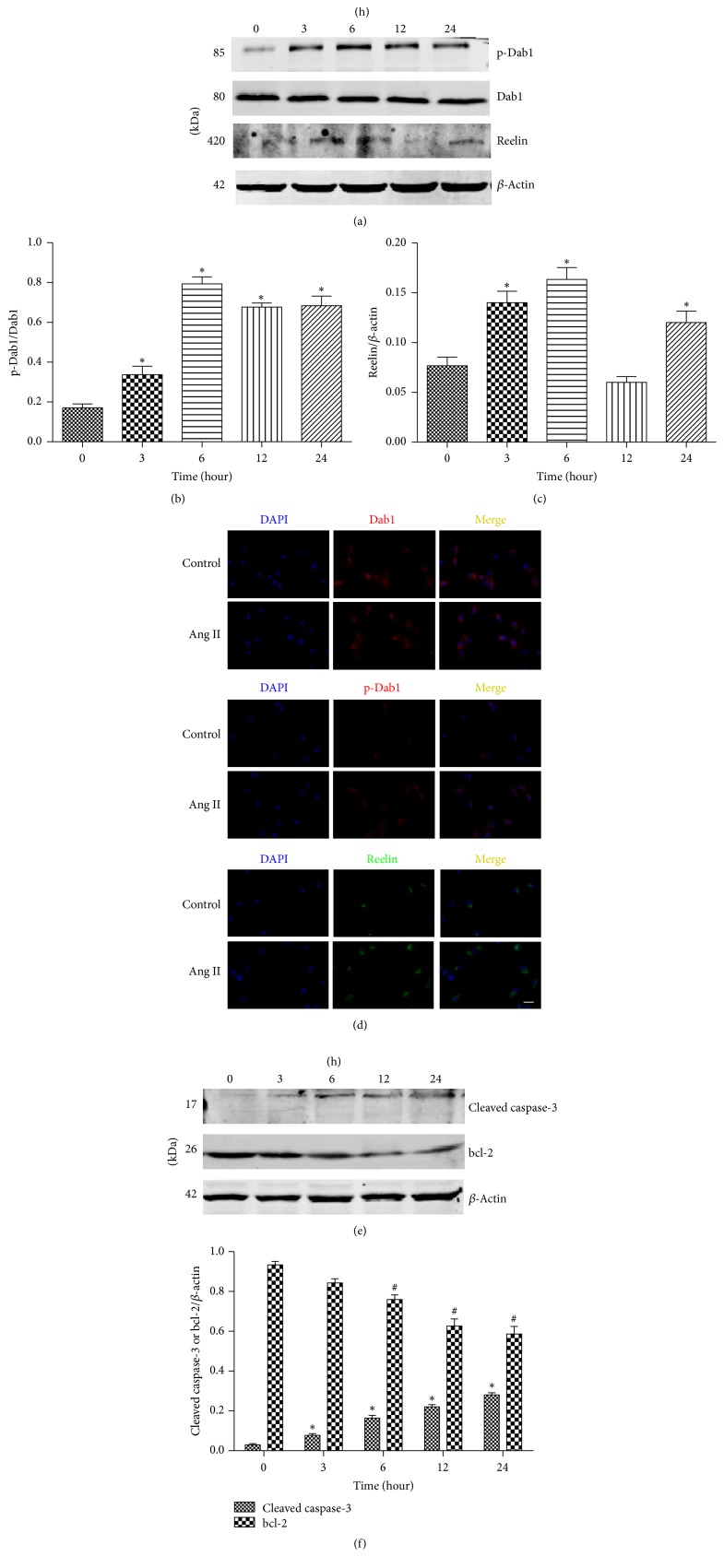
Ang II increases reelin/Dab1 expression in cultured podocytes and induces podocytes apoptosis. (a) Western blotting detected the expressions of phospho-Dab1, Dab1, and reelin in cultured podocytes treated by Ang II (10^−6^ M) at various time points. (b, c) Quantitative analysis of phospho-Dab1 and reelin expression. (d) Immunofluorescence staining of Dab1, phospho-Dab1, and reelin in cultured podocytes stimulated by Ang II (10^−6^ M) for 6 h (original magnification ×400); scale bars, 20 *μ*m. (e) Western blotting detected the expressions of cleaved caspase-3 and bcl-2 in cultured podocytes treated by Ang II (10^−6^ M) at various time points. (f) Quantitative analysis of cleaved caspase-3 and bcl-2 expressions. ^*∗*^*P* < 0.05 versus control group. ^#^*P *< 0.05 versus control group.

To investigate the effect of Ang II on the in vitro expression of reelin/Dab1, we assessed the reelin/Dab1 expression in cultured podocytes. Podocytes were treated with 10^−6^ M Ang II for several time points (0, 3, 6, 12, and 24 h) [10]. Ang II significantly increased the levels of reelin and phosphorylated Dab1 in cultured podocytes in a time-dependent manner (Figures 3(a)–3(c)). Subsequently, immunofluorescence was performed to evaluate the distribution and expression of reelin and phosphorylated Dab1 in Ang II-treated podocytes (Figure 3(d)). Expressions of bcl-2 and caspase-3 were determined to evaluate Ang II-induced podocyte apoptosis. Our data revealed that Ang II exposure significantly increased podocyte apoptosis (Figure 3(e)). The caspase-3 level was also increased after Ang II stimulation in vitro in a time-dependent manner (Figure 3(f)). Conversely, the bcl-2 expression was decreased after Ang II stimulation in podocytes in a time-dependent manner (Figure 3(f)).

### 3.4. Effect of Dab1 on Ang II-Induced Podocyte Apoptosis

**Figure 4:**
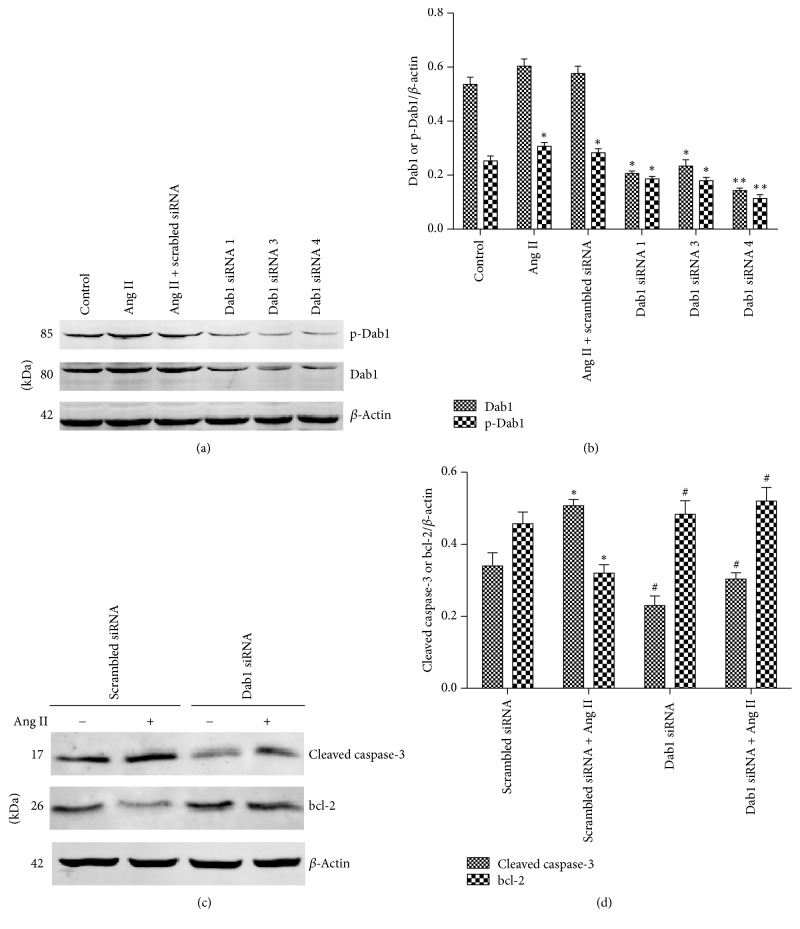
Knockdown of Dab1 diminishes Ang II-induced podocyte apoptosis. (a) Western blotting detected phospho-Dab1 and Dab1 expression in podocytes transfected with Dab1 siRNA. Scrambled siRNA represents a nonspecific nonsilencing siRNA. (b) Quantitative analysis of phospho-Dab1 and Dab1 expression. (c) Western blotting detected expressions of cleaved caspase-3 and bcl-2 in podocytes transfected with Dab1 siRNA in the presence or absence of Ang II. (d) Quantitative analysis of cleaved caspase-3 and bcl-2 expressions. ^*∗*^*P* < 0.05 versus control group. ^*∗∗*^*P* < 0.01 versus control group. ^#^*P* < 0.05 versus podocytes transfected with scrambled siRNA and treated with Ang II.

Podocytes were transfected with Dab1 siRNA to evaluate the role of Dab1 in Ang II-induced podocyte apoptosis. Figures 4(a) and 4(b) show that Dab1 siRNA effectively reduced the phospho-Dab1 and Dab1 expression in podocytes. Western blotting was used to assess podocyte apoptosis. Conversely, the siRNA-triggered Dab1 downregulation prevented Ang II-induced podocyte apoptosis (Figures 4(c) and 4(d)), indicating that Dab1 was involved in the signaling pathways of Ang II-induced podocyte apoptosis.

### 3.5. Effect of Dab1 siRNA on Ang II-Induced p38 and ERK Pathways in Podocytes

**Figure 5:**
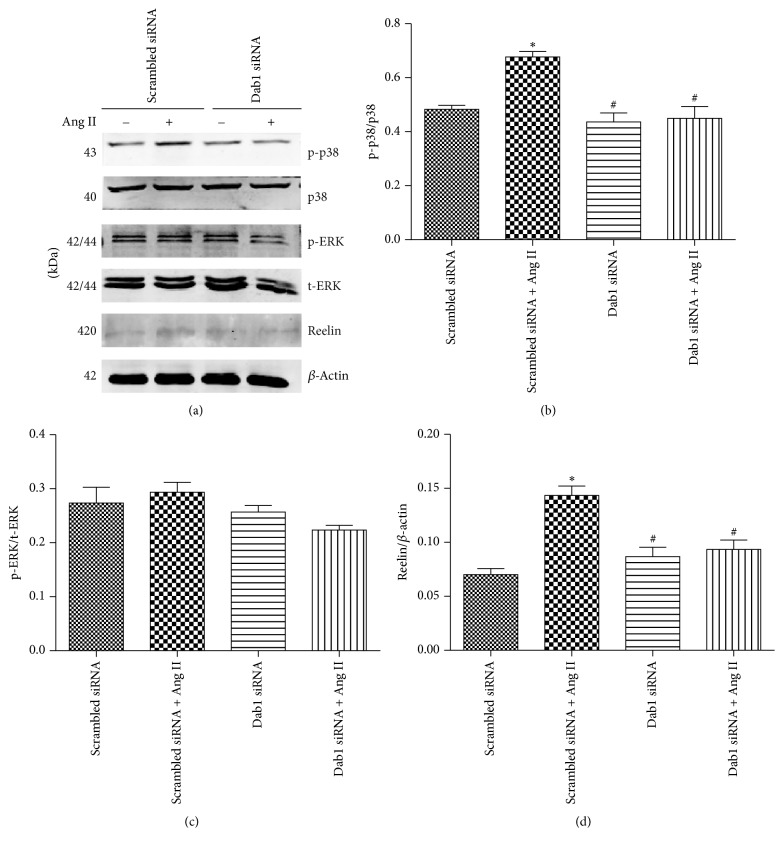
Knockdown of Dab1 affects the MAPK signaling pathway and reelin expression in Ang II-treated podocytes. (a) Western blotting detected phospho-P38, phospho-ERK, and reelin expression in the podocytes. (b–d) Quantitative analysis of phospho-P38, phospho-ERK, and reelin expression. ^*∗*^*P* < 0.05 versus control group. ^#^*P* < 0.05 versus podocytes transfected with scrambled siRNA and treated with Ang II.

It is well known that p38 and ERK pathways participate in regulation of cellular proliferation, differentiation, apoptosis, and so on [17]. Therefore, we evaluated whether p38 and ERK were involved in Ang II-induced podocyte apoptosis when Dab1 was suppressed by siRNA (Figure 5(a)).  Figure 5(b) reveals that Ang II promoted phosphorylated p38 level, but the induced phosphorylation of p38 was diminished by Dab1 siRNA. However, the ERK expression was not affected by Dab1 siRNA (Figure 5(c)). Moreover, knockdown of Dab1 decreased the Ang II-induced upregulation of reelin (Figure 5(d)).

## 4. Discussion

In the present study, we found that Ang II played an important role in the process of proteinuria and podocyte injury. Our previous studies have confirmed the effects of Ang II on podocyte apoptosis and albuminuria [10–12], but the underlying mechanism remains largely unexplored. Here, we reported that Dab1 contributed to Ang II-induced podocyte apoptosis via p38 signaling pathway.

As a secreted glycoprotein, reelin is expressed in Cajal-Retzius cells and granule cells in the cerebellum and olfactory bulb [18, 19]. Reelin plays an important role in the development and maturation of central nervous system, such as neuronal migration, neuronal excitability, and dendritic morphology. Binding of reelin to its receptors VLDLR and/or ApoER2 induces the phosphorylation of adaptor protein Dab1 by the Src family kinases Src and Fyn [20, 21]. Therefore, reelin regulates neuronal development through Dab1 activation. However, the reelin/Dab1 signaling pathway and its role in tissues other than the brain are still poorly understood. In the present study, reelin and Dab1 were detected in kidney of rats and cultured mouse podocytes. Moreover, Ang II facilitated the reelin expression and Dab1 phosphorylation in vivo and in vitro, indicating that reelin and Dab1 were involved in kidney disease and podocyte injury. Consistent with the previous studies, Ang II induced apoptosis of cultured podocytes in a time-dependent manner. However, the siRNA-triggered Dab1 downregulation inhibited Ang II-induced podocyte apoptosis. These results suggested that Dab1 participated in the signaling pathways in the process of Ang II-induced podocyte apoptosis.

In neural development, phospho-Dab1 promotes neuronal migration and regulates neuronal polarization via activating downstream Crk-C3G-Rap1-cadherin and PI3K-Akt signaling pathways [14]. Crk pathway regulates cytoskeleton, and PI3K-Akt pathway modulates cell survival, proliferation, metabolism, and angiogenesis. Dab1 mutant mice exhibit ataxia, tremors, a reeling gait, and neurons aberrantly positioned in laminated brain structures [22], demonstrating that Dab1 phosphorylation is critical for initiation of intracellular signaling. Recently, Yang et al. [23] have reported that binding of activated protein C to ApoER2 activates Dab1-PI3K-Akt signaling in U937 cells. Furthermore, Dab1 has been implicated in the regulation of cholesterol efflux in RAW 264.7 mouse macrophage cell line [24]. Vázquez-Carretero et al. have reported that Dab1 participates in regulation of intestinal crypt/villus unit dynamics [25]. In addition, Dab1 is involved in regulation of mammary gland development, cartilage and tendon differentiation, odontogenesis, and granulosa cell proliferation in chicken follicle [26–30].

We investigated the effects of Dab1 on the MAPK signaling pathway, which plays a vital role in regulation of cellular proliferation, differentiation, and apoptosis [17], because Dab1 can regulate cell survival and proliferation. In our study, knockdown of Dab1 could inhibit proapoptotic effects of Ang II. Subsequently, we explored the p38 and ERK pathway related to cellular apoptosis and proliferation of podocytes.  Figure 5 reveals that Dab1 downregulation inhibited phosphorylation of p38, whereas phosphorylation of ERK was not affected, indicating that Dab1 was involved in Ang II-induced podocyte apoptosis via p38 pathway. Our previous study has confirmed that Ang II induces podocyte apoptosis through p38 MAPK pathway [31]. Recently, Gu et al. [32] have found that olmesartan decreases albuminuria in diabetic nephropathy through inhibiting Ang II/p38/SIRT1-caused podocyte apoptosis. Interestingly, the reelin expression was decreased in podocytes when Dab1 was knocked down. We did not detect the reelin expression in culture medium (data not shown). It is necessary to explore how Dab1 affected the reelin expression in podocytes in future studies.

## 5. Conclusions

Our data showed that Dab1 and reelin could be expressed in rat kidney and cultured mouse podocytes. Podocytes and rats treated with Ang II displayed increased phospho-Dab1 and reelin expression. Ang II could induce apoptosis of cultured podocytes, and the knockdown of Dab1 by specific siRNA inhibited proapoptotic effects of Ang II. Furthermore, Dab1 contributed to Ang II-induced podocyte apoptosis via p38 signaling. Therefore, the Dab1-p38 signaling pathway might be a novel candidate therapeutic target in order to decrease podocyte apoptosis in chronic kidney diseases.
